# Development of an ecological, immersive approach to socio-moral reasoning assessment in virtual reality

**DOI:** 10.3389/fpsyg.2026.1648238

**Published:** 2026-03-26

**Authors:** Erika Neveu, Julia Salles, Isabelle Roy, Evelyn Vera Estay, Julian J. Dooley, Miriam H. Beauchamp

**Affiliations:** 1 Department of Psychology, University of Montreal, Montreal, QC, Canada; 2 Escuela de Psicología, Pontificia Universidad Católica de Chile, Santiago, Chile; 3 FINLA Psychological Services, Athlone, Ireland; 4 Sainte-Justine Hospital Research Center, Montreal, QC, Canada

**Keywords:** adolescence, assessment, digital health, moral reasoning, neuropsychology, social cognition, virtual reality

## Abstract

Social problems affect many children and adolescents, especially those with neurodevelopmental disorders, mental health issues, or social disadvantage. In order to identify children at risk for poor social competence, it is important to be able to document putative difficulties in underlying social cognitive functions, such as their ability to reason and make appropriate decisions when faced with social and moral conflicts, an ability referred to as socio-moral reasoning (SMR). Immersive, dynamic digital health technologies, such as virtual reality (VR), show potential for optimizing assessment of social cognition because they provide features that enhance ecological value. However, the ecological validity of social cognition tasks is seldom formally assessed. The objective of this study was to (1) present detailed information on the design and development of a SMR VR task to support its *a priori* ecological validity and (2) quantitatively test two subcomponents of ecological validity: verisimilitude and veridicality. Sixty-two typically developing adolescents completed the Socio-Moral Reasoning Aptitude Level task-VR version (So-Moral-VR) and questionnaires as indicators of verisimilitude (Simulator Sickness, Presence, Immersive Tendencies), veridicality (Behavior), and social desirability. The results indicate that participants were frequently immersed in the task and perceived realism, had low levels of cybersickness, and a moderate sense of presence, supporting ecological validity through verisimilitude. In addition, a significant correlation was found between SMR and prosocial behavior, suggesting task veridicality. Social desirability effects were low. Taken together, the design principles and quantitative analyses of verisimilitude and veridicality suggest that the So-Moral-VR task demonstrates promising ecological validity and support its use as a quantitative measure of SMR. The task has potential applications in both research and in clinical settings for identifying youth with social cognition difficulties. Future work could extend its validation to clinical populations at risk for poor social competence.

## Introduction

Social problems, including bullying, peer rejection and social isolation, affect between 10 and 30% of children and adolescents, with this figure rising to 40–50% in populations with additional risk factors, such as neurodevelopmental disorders, mental health problems, chronic illnesses, brain injuries and social disadvantage (Biswas et al., 2020; Asher and Coie, 1990; Lim et al., 2023; Abregu-Crespo et al., 2024). Social competence is underpinned by social cognition, a set of cognitive abilities that determine perception, understanding, interpretation and response to the mental and emotional states of others and their behavior (Beauchamp and Anderson, 2010; Tremblay et al., 2023). It is essential in clinical, academic, and community contexts to be able to derive a comprehensive portrait of youth social abilities and to identify individuals at risk for social problems and provide guidance for remediation.

Sound social cognition is particularly important for supporting quality friendships and interactions during adolescence, when peer relationships become increasingly complex, new social roles emerge, and independence is gained (Adams et al., 2006; Goosby et al., 2013). As adolescents encounter more decision-making opportunities, their socio-moral reasoning (SMR) becomes more complex. SMR refers to the ability to analyze and evaluate social situations while considering moral criteria to make judgments and respond appropriately to social conflict and conventions (Adams et al., 2006; Gibbs, 2019; Moll et al., 2005; Gibbs et al., 2013). Poor SMR is associated with maladaptive behaviors such as aggressivity, delinquency, and bullying (Laible et al., 2014; Perren et al., 2012; Van Vugt et al., 2011), while better SMR is associated with prosocial behaviors such as helping others (Laible et al., 2014). SMR evaluation in adolescents is therefore an important component of psychological and neuropsychological assessment to identify social cognition difficulties.

Despite the importance of quantifying and qualifying SMR, assessment tools are limited (Martí-Vilar et al., 2023) and existing tools present methodological challenges and raise concerns about their suitability for pediatric and clinical populations. These challenges include: (1) unrealistic scenarios that fail to reflect familiar experiences, particularly for the pediatric population; (2) third-person and/or in written presentations that reduce emotional engagement and motivation; (3) written scenarios that are time-consuming and depend on adequate reading, attention, abstraction, and working memory skills; (4) scenarios (written or visual) that inadequately represent the complexity of real-life social interactions, such as emotions, non-verbal communication, and the dynamic nature of social interactions (Martí-Vilar et al., 2023; Beauchamp, 2017; Crowe et al., 2021). A systematic review conducted by Martí-Vilar et al. (2023) identified 21 assessment tools for measuring moral processes. However, most of these tools (86%) were quesionnaires, which present several of the above limitations, in particular, the need for adequate reading and attentional skills. Moreover, questionnaires fail to capture the complexity of real-life social interactions, in addition to being prone to subjectivity and social desirability (Crowe et al., 2011; Frankel and Feinberg, 2002). These limitations are all related to a common construct: ecological validity – that is, the extent to which a task reproduces the cognitive and social demands of real-world situations (Franzen and Wilhelm, 1996). The closer an assessment mirrors real-world conditions, the greater its ecological validity (Sbordone, 1996).

Ecological validity comprises two key components: verisimilitude and veridicality (Franzen and Wilhelm, 1996). Verisimilitude refers to how closely a task mirrors the cognitive demands of everyday situations. Veridicality reflects the extent to which test scores relate to other measures that are predictive of real-world task performances (e.g., behavior) (Spooner and Pachana, 2006; Parsons, 2015). Chaytor and Schmitter-Edgecombe (2003) examined both components (verisimilitude and veridicality) in an analysis of 18 neuropsychological assessment tools and concluded that moderate ecological validity was present overall. However, most studies focused on either verisimilitude or veridicality in isolation, underscoring the need for more comprehensive ecological validation of neuropsychology assessment tools. Among 35 standardized cognitive, psychological, and functional measures designed with ecological validity in mind (Rabin et al., 2007), Olson et al. (2013) found that only six were suitable for children, highlighting a particular need for more ecologically valid pediatric neuropsychology tools.

Virtual reality (VR) has the potential to enhance ecological validity in social cognition assessment as it allows the creation of realistic, interactive and immersive virtual environments in controllable and replicable settings (Borgnis et al., 2022). Moreover, VR provides a feeling of ‘presence’ [i.e., “the subjective experience of being in one place or environment, even when one is physically situated in another” (Witmer and Singer, 1998)], which can in turn generate thoughts, reactions, and emotions that reflect those experiences in a real-life situation, creating a more realistic experience, and supporting valid social cognition assessment (Pan and Hamilton, 2018; Pan and Slater, 2011; Tarr and Warren, 2002). However, VR can be challenging to use in clinical setting, especially in pediatric populations (Roy et al., 2024). Therefore, it is important to thoroughly assess such tools in typically developing individuals before considering implementation in clinical populations.

Neveu et al. (2025) conducted a systematic review of VR tools for social cognition assessment. Among 59 tools meeting the review criteria, only one was identified as specifically targeting SMR in the pediatric population: the So-Moral-VR task (Morasse et al., 2021). In addition, the review findings showed that while many authors argue that VR can enhance the ecological validity of social cognition assessments, only a small portion of studies formally evaluate this characteristic, highlighting the need for more systematic ecological validation of assessment tools (Neveu et al., 2025).

Given the need to optimize the association between neuropsychological tasks performance and real-world functioning – that is, to enhance ecological validity—as well as the potential of VR-based assessment tools to address some of the limitations of more traditional tools (e.g., paper-and-pencil, questionnaires, static stimuli), novel VR socio-cognitive assessment tools should be evaluated in terms of both their verisimilitude and veridicality. The So-Moral-VR was designed with ecological value in mind; however, its ecological validity has not yet been formally assessed (Morasse et al., 2021). The aim of the present study was therefore to: (1) provide information about the design and development of the task in order to qualitatively reflect on its verisimilitude; (2) present empirical data from a study on user experience, specifically sense of presence, cybersickness, and immersive tendencies, as quantitative indicators of verisimilitude; (3) report correlations between SMR performance (So-Moral-VR scores) and three measures that are predictive of social functioning, in order to evaluate the veridicality of the task; (4) verify whether participants are prone to social desirability bias when responding to the task.

## Design and development of the So-Moral-VR task

### Original computer-based task and content development

**Figure 1:**
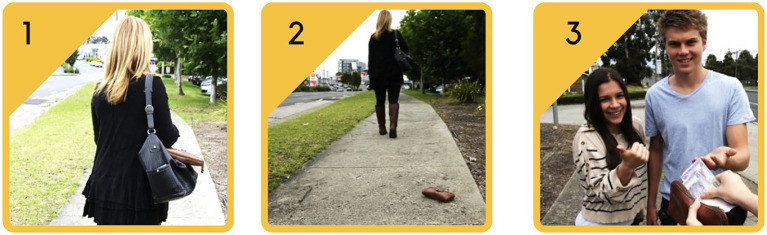
So-Moral Computer Task. This figure represents an example of one of the dilemmas presented to users in the So-Moral Computer Tasks. Here three pictures are presented to show a woman dropping her wallet.

The So-Moral-VR was developed as a variation of an existing computer-based tool extensively tested in a series of validation and clinical studies (Dooley et al., 2010; Chiasson et al., 2017; Vera-Estay et al., 2016; Beauchamp et al., 2019; Beauchamp et al., 2013; Garon et al., 2018a,b). The computer-based task includes between 10 and 19 socio-moral dilemmas, presented on a computer screen as a three static picture narrative of realistic, everyday social situations that require decision-making (see Figure 1) (Dooley et al., 2010). The scenarios portray child actors playing out various social scenarios representing conflicts centered on the moral domain (e.g., concerns with justice, welfare-harm, and rights) according to Social Domain Theory (Turiel, 1983) and the dilemmas were generated to include a range of moral values and social norms.

The dilemmas are presented in the first-person perspective (subjective point of view) (Dooley et al., 2010). The decision to use the first-person perspective was based on a number cognitive, neural, and design considerations. On one hand, the third-person perspective presents a form of indirect questioning that can be useful for avoiding social desirability (because thinking about what someone else should do is less confronting than admitting what one would really do) (Fisher, 1993). This distance from the social problem has also been associated with better reasoning because it encourages perspective taking and compromise (Grossmann and Kross, 2014). On the other hand, this standpoint introduces other issues that make it inadequate for measuring an individual’s own reasoning, decisions, and behavior. For example, functional neuroimaging evidence shows that different brain regions and networks are activated based on whether stimuli are presented in the first- versus the third-person perspective (Boccia et al., 2017; Avram et al., 2014). Relatedly, cognitive and socio-cognitive demands also differ, as shown by the reduced empathic response associated with third-person moral reasoning (Avram et al., 2014). Furthermore, the third-person perspective is associated with actor-observer bias. Defined as the tendency to explain our own actions by the situation, but other people’s actions by their personal traits, this bias can affect moral evaluation and does not accurately represent the processes used in real-life (Avram et al., 2014). Finally, the first-person perspective increases personal and emotional involvement (Orobio de Castro et al., 2002). Thus, to ensure that the task recruited cognitive and neural networks comparable to those engaged during real social interactions—and to mirror real-world experiences as closely as possible—we employed a first-person perspective to enhance ecological validity.

A pilot study by Dooley et al. (2010) provides details on the development of the original computer version of the task including data on familiarity, enjoyment, engagement and comprehension, which are also indicators of ecological validity. All dilemmas were designed to take place in everyday contexts that are meaningful to adolescents. The dilemma familiarity measure indicated that 80% of adolescents reported that they, or someone they knew, had encountered similar situations in real life. Comprehension was also measured to ensure the dilemmas could be understood with no accompanying text, thus precluding the necessity of high-level reading or attention skills (Dooley et al., 2010). Ninety-six percent of participant indicated that they understood the dilemmas, suggesting that they were accessible to most youth (Dooley et al., 2010).

### So-Moral scoring system

One of the main objectives of developing the So-Moral task was to provide a quantitative SMR score, as this can lead to the creation of standardized norms, a practice at the core of psychological and neuropsychological assessment, and the comparison of SMR maturity levels across individuals (e.g., clinical populations, age groups). After viewing a dilemma, participants are asked whether they would engage in the proposed action and why they would or would not do so. For example, one dilemma shows a classmate asking for the answers to an exam. The participant (or user) is then asked, “Would you do this?” (i.e., give your answers to your friend) and “Why?.” The answers are recorded, transcribed, and coded to generate two scores. First, the socio-moral decision-making score (SMDM) is based on the dichotomic response (“yes” or “no”) to the first question. Second, the SMR maturity score is based on the answer to the second question (Why?). Verbatim justification responses are recorded and coded using a 5-level scoring system adapted from the cognitive-developmental approach (Gibbs, 2019; Dooley et al., 2010; Kohlberg, 1981). This distinction in scores aligns with theories arguing that moral judgment tends to be intuitive, whereas moral reasoning is constructed after the judgment has been made to justify it (Haidt, 2001). Developmental stages of moral reasoning have been adapted to fit the social nature of the dilemmas in the So-Moral task and consist of the following: (1) centration and authoritarian-based consequences; (2) egocentric/pragmatic exchanges; (3) interpersonal focus; (4) societal regulation; and (5) societal evaluation.

Transition stages (1.5, 2.5, etc.) are used to account for answers that provide elements of two consecutive reasoning stages. These transition stages are attributed when the response is not developed enough to belong to the higher level. For example, in the dilemma about whether a person would share their exam answers with a peer, a participant might say: “Yes (I would share my answers), because everyone has their own difficulties and the school system is trash” and obtain a score of 4.5 because they provide elements of social regulation (4) and a partial response for social evaluation (5), but without fully explaining their thinking regarding the latter (missing information regarding why it is “trash”). When an answer combines elements of two non-consecutive stages, the higher level is attributed. That is, a participant might provide a high-level component in their answer, but also include additional reasons that would score at a lower level if given in isolation. For example, for the same dilemma, a participant might answer “*No (I would not share my answers), because the teacher is present and I think it goes against the goal, for example, I have studied and it is not good after all that to give my work to someone, while they have probably not done much. Studying is an accomplishment and it allows you to have good grades and the other person has not studied so it’s their problem. But for sure I will explain it to the person, even if they will not agree with my decision*”. This participant would score 5 because their answer shows a societal evaluation that is developed enough to obtain this level, even though there are elements of the lower (1) centration and authoritarian-based level (reference to the teacher being present). The total moral maturity score is therefore the total of the SMR reasoning scores obtained for each dilemma.

In sum, the task design and scoring, grounded in a cognitive-developmental framework, assumes that more mature reasoning is characterized by greater cognitive flexibility and abstraction. It enables individuals to “decenter,” considering not only the personal consequences of a decision, but also its broader impact on societal functioning. For this reason, the system assigns the score based on the most mature reasoning expressed, even when the response also includes elements of simpler reasoning. Because the justification is articulated in the first person, it allows underlying socialization processes to emerge. While this qualitative richness may diminish when the tool is applied in quantitative research, its clinical and intervention potential lies precisely in this personalized dimension. Spontaneous responses enable young people to integrate situated social knowledge into their reasoning (e.g., “in my school or in my neighborhood X is not accepted”), and to articulate arguments drawing on deontological, utilitarian, care ethics, and other moral frameworks. Most importantly, it encourages them to reflect on and weigh these considerations in their decision-making. This process can help identify tendencies and biases in reasoning and could progressively guide individuals toward more socially adapted forms of moral reasoning and decisions.

### Selection of dilemmas for the VR version of the So-Moral task

#### Dilemmas, choices and modification process

The VR version was developed with the goal of further enhancing the ecological validity of the original task through the addition of dynamic and multimodal elements (e.g., character movement, sound) and immersive 3D environments (Sato and Yoshikawa, 2007; Paiva-Silva et al., 2016; Rymarczyk et al., 2011; Sato et al., 2008). To do so, a team of neuropsychologists and VR developers/programmers identified and selected the most appropriate dilemmas from the original So-Moral task to ensure the feasibility of transitioning from a 2D, static, computer-based format to an immersive VR environment. The VR task also presents the dilemmas in the first-person point of view, for the reasons explained in the previous section, as well as to elicit a greater sense of presence and involvement in the task (Kallinen and Salminen, 2007; Slater et al., 2010; Parsons, 2015).

#### Elements influencing the decision to include or modify a dilemma

Incorporating virtual hands generally diminishes sense of presence, because the mismatch between a virtual and real hands makes users more aware of the artificial nature of the environment (Kokkinara et al., 2015). Therefore, dilemmas requiring obvious hand use (e.g., picking something up) were excluded. Other dilemmas were excluded due to the complexity of interactions or details to be implemented in VR (e.g., Kiss a friend’s partner). Some dilemmas were prioritized or adapted to optimize the development process when virtual objects or locations were readily accessible via banks of VR images or previously developed environments (e.g., entering a *movie theater* without paying became leaving a *restaurant* without paying). Task duration was also considered with the goal of limiting to a maximum of 20 min to be appropriate for clinical and research contexts and to reduce cybersickness effects, while still including enough dilemmas to be able to draw meaningful conclusions about SMR performance (Kim et al., 2023).

#### Final content of the VR task

**Figure 2:**
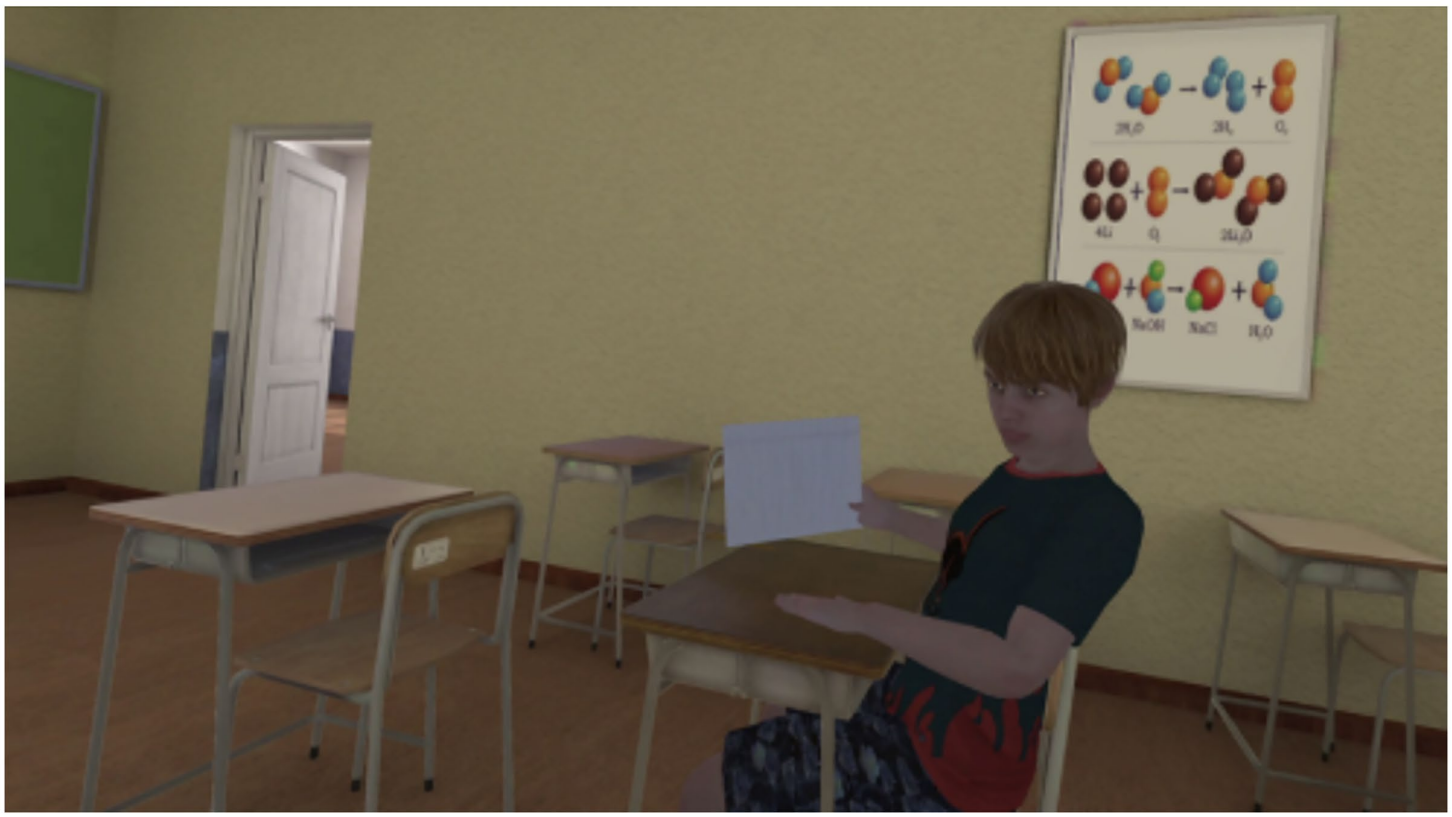
So-Moral-VR task. This figure represents an example of one of the dilemmas presented to users in the So-Moral-VR task. Here we see the user’s friend asking for the exam answers.

Following this process, nine dilemmas were included in the VR version, resulting in a task duration of approximately 15 min (Figure 2). A practice scene was added at the beginning to allow participants to become comfortable with the technology and practice navigating and interacting with VR, and to familiarize themselves with the first-person point of view, thus further ensuring the validity of their responses.

### Storyline and optimization of sense of presence in the virtual environment

In contrast to the computer-based version which presents the dilemmas in no particular logical order, the VR task was designed as a storyline with a sequential, logical presentation of the dilemmas to ensure that sense of presence is not disrupted after each dilemma is completed. Users begin in a virtual house, simulating the start of a typical day and in line with the goal of recreating everyday experiences in an adolescent’s life. The user picks up their mobile phone and leaves their house to complete the dilemmas, moving from one familiar setting to another (house to school, through the town, and into a restaurant, for example).

The original task required an examiner to elicit responses to the dilemmas by directly asking the user verbal questions. However, the use of an external individual in virtual reality can break the immersion phenomenon and sense of presence, thus reducing ecological validity (verisimilitude). In addition, this can introduce social desirability bias on the part of users, because they may feel pressured to answer in socially expected ways, potentially compromising the validity of the task (Oh et al., 2019; Palombi et al., 2023). To circumvent these problems, at the beginning of the task, the user picks up their mobile phone from a coffee table in their virtual house and keeps it with them throughout the whole assessment. The mobile phone is used to deliver the assessment questions using pre-recorded audio stimuli, as if the participants were speaking to someone on a call. This design feature was included with the goal of reducing social desirability bias by limiting the presence of the examiner and ensuring immersion is maintained, thus tending towards a self-administered task (Nederhof, 1985).

### VR environment development and platform

To induce the question coming from the phone, a “Wizard of Oz” effect (i.e., a behavior that seems to come from a virtual character or environment in the VR assessment, but is actually driven by a human controlling it) was used by programming specific keyboard keys for the examiner to trigger the questions within the virtual environment at appropriate moments without needing to directly interact with the user, allowing for more control and flexibility, and better human-computer interaction and user experience (Pan and Hamilton, 2018). The initial version of So-Moral-VR (Morasse et al., 2021) was developed using *Virtools®*, but the task was subsequently reprogrammed and upgraded to *Unity®*. Platform choices were based on the developers’ recommendations, available resources at the time, and technological compatibility (e.g., *Virtools®* was not compatible with *Oculus®* devices, which emerged over the course of development and initial validation). The transition from *Virtools®* to *Unity®* also enabled enhancements in visual stimulus quality.

### Navigation and interaction components

The VR task also enhances ecological value by incorporating interaction, navigation, and audio elements within an immersive virtual environment. These additions make the stimuli more dynamic and realistic (Paiva-Silva et al., 2016; Rymarczyk et al., 2011; Sato and Yoshikawa, 2007). The VR task was developed to be experienced using six degrees of freedom (6DoF) headsets to maximize immersion and interactivity. A 6DoF navigation allows users to move freely in three-dimensional space—forward/backward, up/down, left/right—as well as rotate around three axes of head movements (pitch, yaw, and roll). To move within the virtual environment users must press a button on the joystick to move forward and look in the direction they wish to go. This navigation mode contributes to ecological validity, as people typically look in the direction they intend to move. This approach also mirrors real-world cognitive demands, requiring users to actively produce movement aligned with their intended direction. The task navigation mode was designed to be as immersive as possible while maximizing user control, to minimize the risk of cybersickness (Davis et al., 2014). Teleportation, a navigation mode used often in VR games, is not the default mode in So-Moral-VR, as continuous movement facilitates control during the assessment (though it is possible to activate the teleportation navigation mode using the joystick). Further, users can remain seated throughout the task to facilitate transferability to clinical settings—where physical space is often a limitation—and to further reduce the risk of cybersickness (Kim et al., 2023).

To prevent users from becoming disoriented in the complex VR environment and to keep the task duration as short as possible, *wayfinding markers* (small indicator lights) were added to guide them through the virtual city, and certain paths were intentionally blocked. These lights were designed to be subtle enough to preserve the sense of presence. A common feature in many video games, wayfinding markers are familiar to many users, unlikely to disrupt immersion and flow, and can help to reduce cognitive loads, negative affect and feelings of pressure (Irshad et al., 2021). Although these markers may seem to affect the ecological validity of the task—since participants are not entirely free to explore—they actually reduce the cognitive demands of deciding where to go and reflect a real-world parallel: in daily life, individuals usually have a goal or destination in mind and do not wander aimlessly. Hence, the use of wayfinding markers enables users to focus on the primary task, which is to respond to the moral dilemmas, rather than on navigation or exploration. Further, such features can reduce cognitive load and avoid attentional distractors which may support task validity in individuals with attentional difficulties.

### Audio and visual elements to enhance attention and realism

Sound elements were included to ensure users direct their attention to the appropriate locations in the virtual environment, thereby supporting spatial attention (the ability to selectively pay attention to relevant locations in the visual field) (e.g., dog barking). These audio cues also enhance engagement, realism, sense of presence and task validity (Cooper et al., 2018; Rogers et al., 2018; Moore and Fallah, 2001). The audio clips included in So-Moral-VR are intentionally short in order to closely match the original version of the task (which does not include sound or words) and ensure comparability, a design consideration supported by Morasse et al. (2021).

The locations depicted in the computer version were reproduced in VR to be as detailed as possible (e.g., the store is fully stocked and includes product details) to ensure familiarity and closely resemble real-life scenarios and enhance presence (Abramson et al., 2021; Cerda et al., 2021; Pouke et al., 2019). Some complex movements were created using motion capture technology to allow more precise and fluid avatar movements (e.g., throwing a rock), further increasing immersion and realism.

### Character diversity

Efforts were made to include avatar diversity within the environment such as representations of different ethnicities, gender, and age groups. This design feature was included to enhance inclusion, cross-cultural validity and realism. However, it is important to acknowledge that use of the task in certain groups, regions, or cultures may require further adaptations to accurately reflect regional realities (Reyes et al., 2024).

In sum, a number of design and development choices were made to ensure the ecological validity of the So-Moral-VR including storyline, scripts, visual, dynamic and auditory stimuli. These choices support the verisimilitude of the task by qualitatively demonstrating how the So-Moral-VR task closely reproduces adolescent daily life and social situations. An empirical study was then conducted to quantitatively assess ecological validity in the So-Moral-VR.

## Methods

### Study design

Two cross-sectional study recruitment phases were conducted with the same participant criteria. Some of the assessment measures differed between the two phases, but a core set of measures allowed pooling of the data for certain variables. Exact samples for each measure are reported in the analyses.

### Participants

Sixty-two participants aged between 11 and 25 years (41.9% male, Mage = 16.72, SD = 2.87) were recruited through convenience sampling via flyers posted in recreation and community centers, social media, and high schools, colleges, and universities. Inclusion criteria were: (1) aged between 11 and 25 years and (2) fluent in French or English. Exclusion criteria were: (1) a diagnosed (as reported by the primary caregiver or the participants themselves) neurodevelopmental, acquired, or psychiatric condition such as attention deficit hyperactivity disorder, learning disabilities, schizophrenia, generalized anxiety disorder, autism spectrum disorder, or epilepsy; (2) prior acquired brain injury leading to a visit to the emergency department (e.g., concussion, traumatic brain injury, stroke); and (3) any cardiac condition. The study was approved by the local Research Ethics Committee. All participants provided written informed consent prior to participation (for participants under the age of 14 years, consent was provided by parents or legal guardian). Participants received a modest monetary compensation for participating. Participants completed an in-house demographic questionnaire to document medical history, educational level, and ethnicity.

### Socio-moral reasoning measure

#### Socio-Moral Reasoning Aptitude Level-VR version

The So-Moral-VR was developed and rendered using Unity version 2019.4.35f1. Participants are presented with 9 different social situations depicting a moral conflict in a dynamic virtual environment as a continuous story, during which the participant must follow light indicators through the virtual environment to navigate from one dilemma to another. Participants receive a virtual phone call through which an integrated voice provides instructions at the beginning of the task and generates the questions related to the main variables of interest, socio-moral decision-making (SMDM) and socio-moral reasoning maturity (SMR), after each dilemma. The same scoring process is used in the VR version as for the original, computer-based task (described earlier in the text). Briefly, the aggregate number of morally adapted responses is compiled to obtain the SMDM score, which ranges in the VR version from 0 to 9 points. Participants are then asked to provide a justification for the choice they made. Each participant’s justification is recorded verbatim and subsequently scored according to a standardized coding system based on a cognitive developmental approach (Kohlberg, 1981; Gibbs, 2010; Turiel, 1983; Dooley et al., 2010). The developmental stages of moral reasoning have been adapted to fit the social nature of the dilemmas in the So-Moral and consist of the following: (1) centration and authoritarian-based consequences; (2) egocentric/pragmatic exchanges; (3) interpersonal focus; (4) societal regulation; and (5) societal evaluation. Transition stages (1.5, 2.5, etc.) are used to account for answers that provide elements of two consecutive reasoning stages and when an answer combines elements of two non-consecutive stages, the higher level is attributed. The total moral maturity score for the VR version ranges from 0 to 45 points.

### Measures of verisimilitude

#### Immersive tendencies questionnaire (ITQ)

This 18-item self-report questionnaire measures the capability or tendency of individuals to be involved or immersed (e.g., “Do you easily become deeply involved in movies or TV drama?”) on a scale rated from 0 (“never”) to 7 (“often”). Four subscale scores are reported: focus (i.e., tendency to maintain focus on current activity), involvement (i.e., tendency to become involved in activities), emotions (i.e., tendency to feel emotions during activities), games (i.e., tendency to play with video game) (Witmer and Singer, 1998; Robillard et al., 2002).

#### Simulator sickness questionnaire (SSQ)

This is a self-report measure of cybersickness symptoms that includes 16 items rated on a scale from 0 (“not at all”) to 3 (“severely”). The two subscale scores, nausea and oculo-motor difficulties, were used to reflect verisimilitude. A higher score corresponds to greater levels of cybersickness symptoms (Kennedy et al., 1993).

#### Presence questionnaire (PQ)

The 7-item self-report *Realism* subscale was completed to evaluate the perceived realism of the virtual environment [e.g., “How natural did your interactions with the environment seem?” rated from 0 (“not at all”) to 7 (“completely”)]. The final score is the average of the responses, with scores from 1 to 3 indicating a negative experience and scores from 5 to 7 indicating a positive experience (Witmer and Singer, 1998).

### Measures of veridicality

#### Strength and difficulties questionnaire (SDQ)

This 25-item self-report questionnaire measures five aspects of behavior (5 items for each subscale), emotional symptoms, conduct problems, hyperactive/inattention, peer relationship problems, and prosocial behavior on a scale from 0 (“not true”) to 3 (“certainly true”). The final score is the sum of the item responses, with higher scores indicating greater behavioral difficulties, except for the prosocial subscale for which higher scores reflect more positive behaviors. The conduct problems, peer relationship problems, and prosocial behavior subscales were chosen *a priori* as indicators of veridicality for the So-Moral-VR (He et al., 2013).

#### Marlowe-Crowne social desirability scale

This 33-item self-report questionnaire assesses the tendency to respond in a socially desirable manner using a dichotomous rating of 0 (“false”) or 1 (“true”). The final score is the sum of the items, with higher scores indicating greater likelihood of socially desirable response bias (Strahan and Gerbasi, 1972).

### Statistical analyses

Statistical analyses were conducted using SPSS software version 29.0. Data were examined for any violation of test assumptions (normality, linearity, and homoscedasticity). Two participants did not complete the VR task due to significant cybersickness symptoms, and one participant completed some questionnaires but did not attend the in-person assessment. Exact sample sizes are presented given slight differences in the administration protocols and missing data. To report on verisimilitude, descriptive analyses were conducted to assess immersive tendencies (Involvement, Focus and Game subscales, *N* = 58; Emotion subscale *N* = 32), cybersickness symptoms (*N* = 31) and realism (*N* = 31). To identify the strengths and weaknesses of the task in terms of verisimilitude, descriptive analyses of specific items from the SSQ (*N* = 31) and PQ realism (*N* = 31) subscales were conducted. Since literature has shown that cybersickness can negatively impact the sense of presence (Riches et al., 2019), a Pearson correlation analysis between cybersickness symptoms and realism was performed (*N* = 31). To support veridicality, Spearman correlation analyses (*N* = 59) were performed to examine the relations between SMR maturity and conduct problems, peer relationship problems, and prosocial behavior. Descriptive analyses of social desirability (*N* = 62) were also conducted to interpret the results in light of potential response bias (Schonfeld et al., 2005). Further Spearman correlations were conducted between social desirability and So-Moral-VR scores (SMR *N* = 59; SMDM *N* = 57), as well as with conduct problems (*N* = 62), peer relationship problems (*N* = 62), and prosocial behavior (*N* = 62), to better understand how social desirability may affect participant responses. Results with *p* < 0.05 were considered statistically significant. The strength of correlations and effect sizes were determined according to Cohen’s criteria (Cohen, 1988).

## Results

### Verisimilitude of the So-Moral-VR task

**Figure 3:**
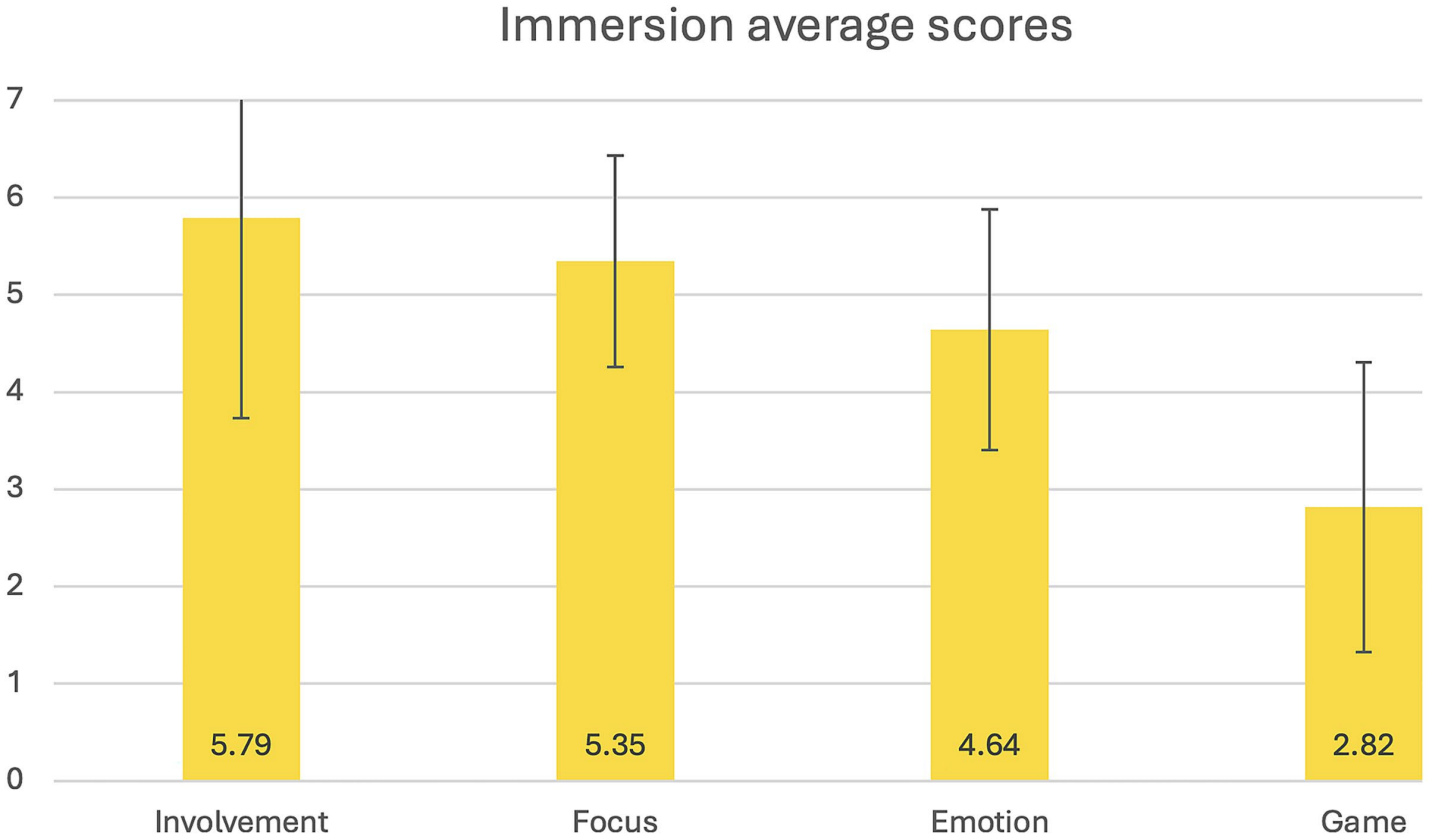
Immersion average scores. This figure represents the average score on all the subscales (Involvement, Focus, Emotions, Game) of the *Immersive Tendencies Questionnaire*.

The overall mean immersive tendencies (ITQ) score was 4.511 (SD = 0.803), which corresponds to a rating between “occasionally” and “often.” Among the subscales, the highest mean score was observed for Involvement, followed by Focus and Emotion, all within the “occasionally to often” range (see Figure 3). The Game subscale showed the lowest score, corresponding to “occasionally” (see Figure 3).

**Figure 4:**
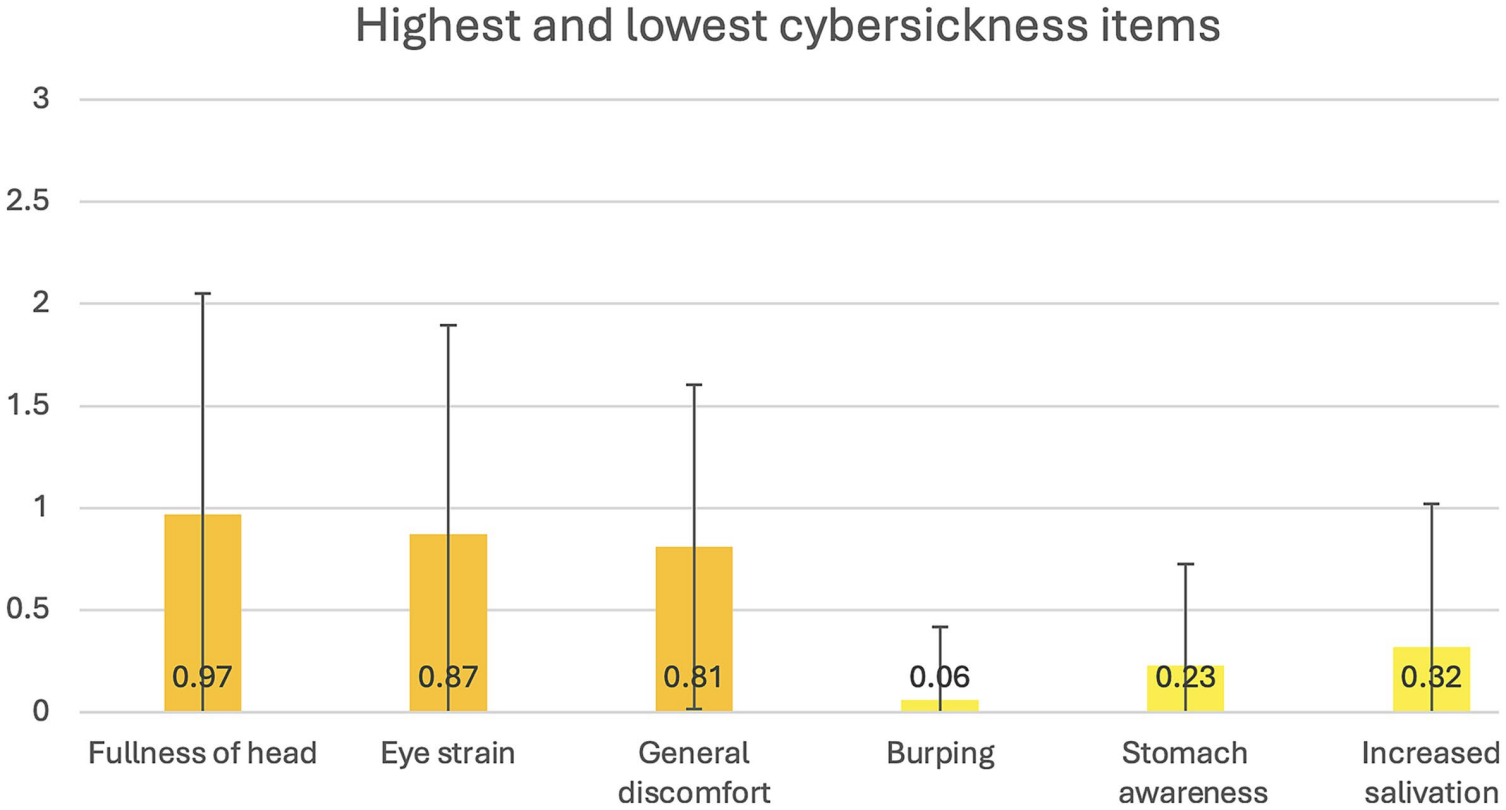
Highest and lowest cybersickness items. This figure represents the average scores on the three highest and lowest rated items from the *Simulator Sickness Questionnaire*.

Two participants did not complete the task due to cybersickness. The mean level of cybersickness symptoms was 0.583 (SD = 0.059), falling between “not at all” and “a little.” The nausea symptom subscale had a mean of 0.484 (SD = 0.599), while the oculo-motor difficulties subscale had a mean of 0.710 (SD = 0.629), both falling between “not at all” and “a little” and reflecting only mild symptom levels. The three highest mean scores on individual SSQ were: “Fullness of head”, “Eye strain” and “General discomfort” (see Figure 4), corresponding to “a little.” The lowest mean scores were reported for “Burping”, “Stomach awareness” and “Increased salivation” (see Figure 4), corresponding to “not at all.”

**Figure 5:**
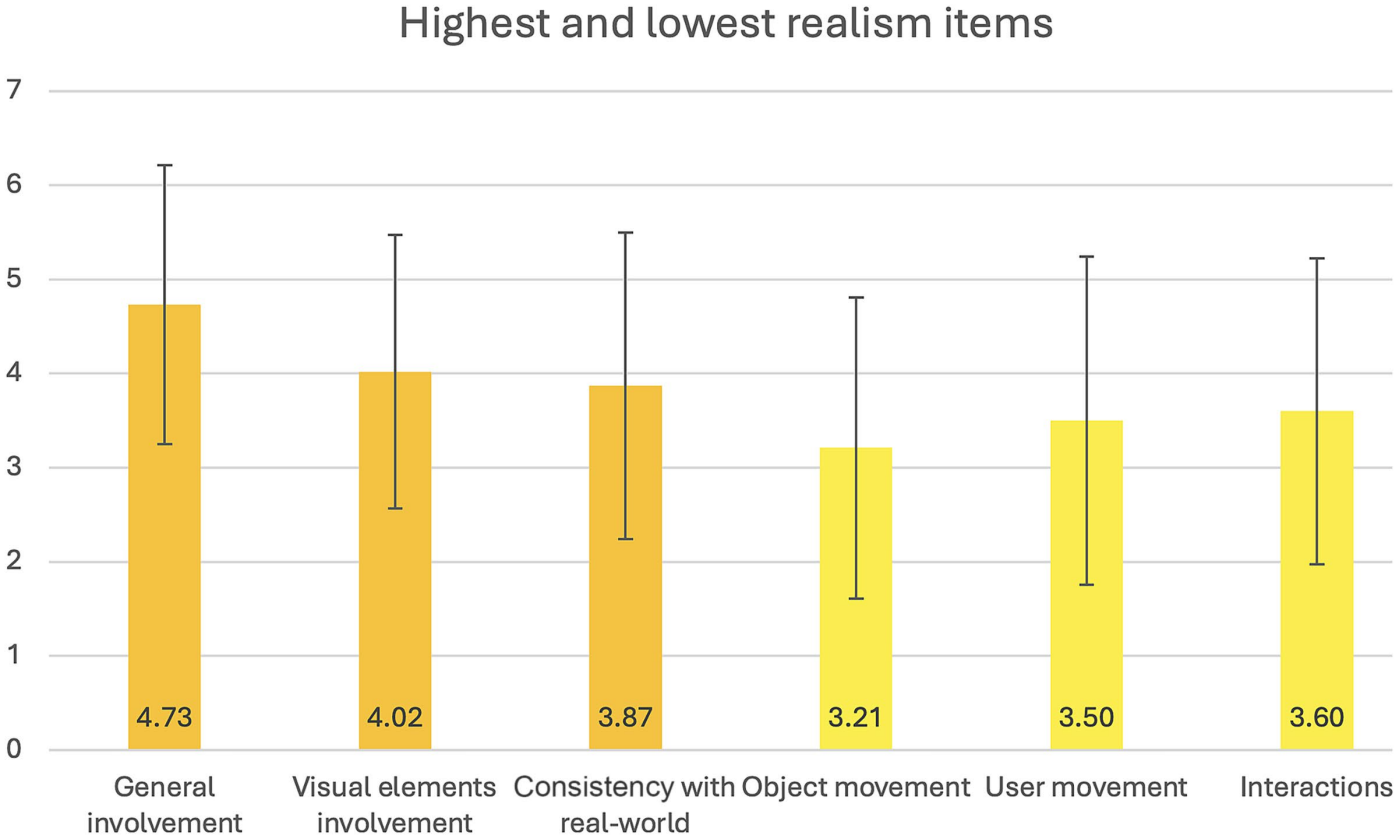
Highest and lowest realism items. This figure represents the average scores on the three highest and lowest rated items from the *realism* subscale in the *Presence Questionnaire*.

The mean realism score on the PQ was 3.80 (SD = 1.184), corresponding to “somewhat” and indicating a moderate sense of presence in relation to the realism of the virtual environment. The highest-scoring items were: “How involved were you in the virtual environment experience?”, “How much did the visual aspects of the environment involve you?” and “How much did your experiences in the virtual environment seem consistent with your real-world experiences?” (see Figure 5), falling, respectively, between “mildly involved” and “completely involved”/“somewhat”, and “completely”/“moderately consistent” and “very consistent.” The lowest-scoring items were: “How compelling was your sense of objects moving through space?”, “How compelling was your sense of moving around inside the virtual environment?” and “How natural did your interactions with the environment seem?” (see Figure 5), corresponding to “moderately compelling”/“borderline.” A moderate and significant association was found between cybersickness symptoms and sense of presence (*R*^2^ = −0.376, *p* = 0.037).

### Veridicality of the So-Moral-VR task

Results showed a statistically significant correlation (*R*^2^ = 0.283, *p* = 0.030) between SMR (*M* = 27.831; SD = 5.847) and prosocial behavior (*M* = 9.113; SD = 1.189), with a small effect size. The associations between SMR and conduct problems (*M* = 2.161; SD = 1.58; *p* = 0.938) and peer relationship problems (*M* = 1.790; SD = 1.621; *p* = 0.896) were not statistically significant. The mean score on social desirability was 16.774 (SD = 5.026), indicating a moderate influence of social desirability on participants’ answers. Social desirability was not significantly correlated with either SMR (*p* = 0.177) or SMDM (*M* = 6.842; SD = 1.031; *p* = 0.689). Further, total SMDM had a mean of 6.842 (of 9 possible points), suggesting that participants do not always provide the most socially desirable answer; 24% of the time they made a decision that is less morally or socially adapted. A small, but statistically significant, correlation was observed between social desirability and conduct problems (*R*^2^ = −0.299, *p* = 0.018). No significant associations were found between social desirability and peer relationship problems (*p* = 0.481), or prosocial behavior (*R*^2^ = 0.246, *p* = 0.054). Given the latter neared the significance threshold with a small effect size, it is possible that the association may emerge in a larger sample.

## Discussion

This study aimed to document the ecological validity of a VR social cognition task, the So-Moral-VR, by providing detailed information on the design and development choices that support verisimilitude, as well as the results of an empirical study including quantitative measures of verisimilitude and veridicality. Description of the design and adaptation features confirm that ecological validity was planned *a priori* by using a first-person point of view, relevant and familiar socio-moral dilemmas that adolescents can relate to, audio stimuli (e.g., dog barking, friends laughing), detailed visual environments (e.g., school, shop) and diversity in avatar appearance (e.g., gender, ethnicity, age). The empirical findings from the study indicate that these design principles successfully led to perceived realism of the task, did not induce significant social desirability bias or cybersickness symptoms, and created a VR measure that is associated with real-life manifestations of prosocial behaviors in typically developing youth. Taken together, the design principles and quantitative analyses of verisimilitude and veridicality suggest that the So-Moral-VR task is associated with several indicators of ecological validity.

### Design principles for ecological validity in So-Moral-VR verisimilitude

Numerous design choices were made during the development of So-Moral-VR to ensure its ecological validity, as described in the Design and Development section, and to ensure that the task closely mirrors the cognitive demands of everyday situations, thus enhancing verisimilitude. The selected dilemmas present a storyline and virtual environments (e.g., shops, school, streets) that are likely to be commonly encountered by adolescents, as demonstrated by Dooley et al. (2010). However, since the dilemmas and virtual environment were created and validated in Canada and Australia, they may be more representative of western, educated, industrialized, rich, and democratic (WEIRD) societies. The relevance of the content may be different in other regional, cultural or socio-economic settings (Reyes et al., 2024), which could affect familiarity and ecological validity and may require trans-cultural adaptations. For example, families with fewer resources may not go to restaurants. One dilemma represents a billiard game, which may not be popular in certain regions. As such, the task is likely to require trans-cultural adaptation to maintain an adequate level of verisimilitude in different socio-cultural contexts (Pinto et al., 2025).

The So-Moral-VR allows limited customization of the environment and avatars, and enhancing these aspects could increase sense of presence, body ownership, emotional presence, engagement and intrinsic motivation, all of which can contribute to stronger ecological validity (Waltemate et al., 2018; Koulouris et al., 2020). The immersive, interactive, and dynamic aspects of VR, along with the use of sound cues likely contribute to reproducing the contextual and cognitive demands of everyday situations, further enhancing verisimilitude (Sato and Yoshikawa, 2007; Paiva-Silva et al., 2016; Rymarczyk et al., 2011; Sato et al., 2008; Cooper et al., 2018; Rogers et al., 2018; Moore and Fallah, 2001). Nonetheless, technological advancements that have occurred since initial task development could offer further technical features that enhance verisimilitude. For instance, using 360-degree videos with human actors could increase the realism of both avatars and virtual environment, potentially offering more nuanced emotional expressions and more natural movement (Barreda-Ángeles et al., 2020). On the other hand, using this technology could also limit avatar and environment customization.

### Quantitative support for verisimilitude in the So-Moral-VR task

The So-Moral-VR task shows verisimilitude in typically developing adolescents (i.e., how closely a task mirrors the cognitive demands of everyday situations) as reflected by four indicators: the participants’ moderate to high ability to feel immersed in the virtual environment, the low incidence of cybersickness symptoms, a moderate level of realism, and the association between cybersickness and realism.

#### Participants’ immersive tendencies

Participants reported enjoying immersive virtual environments, having good concentration during immersive activities and being able to ignore distractions. They also reported a strong tendency to feel emotions and identify with characters in immersive environments. However, they showed low interest in video games, which is not uncommon in non-gamer-specific samples such as this one. Results in this sample nonetheless aligned with results from a validation study of the Immersive Tendencies Questionnaire and expected levels for this population (Robillard et al., 2002).

#### Low cybersickness levels

Two participants (representing only 3% of the sample) could not finish the task due to cybersickness. It is possible that their particular susceptibility to cybersickness may have been due to individual factors, such as sensitivity to motion sickness rather than the task itself. A history of motion sickness is a strong predictor of cybersickness (Kourtesis et al., 2024). Overall cybersickness symptoms were low, which contributes to the validity of the task, as previous studies have shown that cybersickness can negatively impact cognitive performance in VR (Kourtesis et al., 2024). The presence of such symptoms in a VR task can distract users from engaging with dilemmas, reduce their sense of presence in the environment, and impair their performance (Ramaseri Chandra et al., 2022; Riches et al., 2019). As expected, symptoms such as stomach awareness, increased salivation, and burping were not endorsed, consistent with previous findings that these symptoms are generally less prevalent in VR compared to nausea (Cossio et al., 2025). However, somewhat higher scores were observed for symptoms such as fullness of head, eye strain, and general discomfort. Future iterations of the task could address these issues by narrowing the field of view, reducing rapid changes in acceleration and deceleration, foveated rendering, and using image blurring techniques (Hussain et al., 2021; Ang and Quarles, 2023). The minimal cybersickness reported by participants supports the task’s verisimilitude by avoiding sensations and cognitive demands that are not present in real-world experiences and reduce sense of presence (Ramaseri Chandra et al., 2022).

#### Moderate levels of realism

Participants reported a moderate level of realism, suggesting adequate task verisimilitude, but also room for improvement. To elicit the same cognitive, social, and behavioral functions as those involved in everyday situations (verisimilitude), the virtual environment must be as realistic as possible. Participants reported feeling involved and concentrated during the task, suggesting that the key design choices (e.g., virtual environment, storyline, dilemmas) enhanced verisimilitude. This sense of involvement and focus could support engagement and motivation, which are known to influence task performance, particularly in children and adolescents (Borgnis et al., 2022; Pieri et al., 2023). Lack of motivation can compromise assessment validity as responses may no longer reflect the child’s actual abilities (Bertrand et al., 2023; Flaro et al., 2000). Given the So-Moral-VR is immersive and realistic, it could foster greater engagement and motivation, ultimately enhancing both ecological validity and the overall utility of the tool for pediatric use.

Several elements related to the perceived realism of the task (stimuli movement, interaction and navigation) received relatively lower ratings, suggesting an adequate, but improvable level of realism. This could be done through enhancement of movement and interactions: current animations, while functional, could be made more natural through the use of 360-degree videos, which can capture the motion of real actors. Currently, interaction within the task is limited to navigating the virtual environment and picking up a cellphone at the beginning of the task. This limited interactivity may have led to lower ratings and could be enhanced by allowing participants to enact their decisions. For example, if a participant chooses to share exam answers with their friend, they could be required to take action in the virtual environment, hand over an exam sheet and experience the consequences. While interesting, adding this type of interaction would, however, increase task duration, complexity, and the nature of the assessment. It could introduce technical issues and make users more aware of the artificial nature of the environment because of the need to program virtual hands (Kokkinara et al., 2015).

Navigation features should also be considered in relation to realism. Currently, users control their movement using a joystick while remaining seated. Although allowing participants to physically walk through the virtual city might logically enhance realism and the sense of presence, prior research shows that there are no significant differences in immersion between standing and seated VR experiences (Kim et al., 2023). Notably, requiring participants to stand and move around could place limitations on the clinical applicability of the task because it may not be practical in clinical settings (e.g., psychologists’ offices), where space is limited.

#### Association between cybersickness and realism

Cybersickness was negatively associated with realism. Participants who reported higher levels of cybersickness symptoms tended to score lower on the realism scale, which is in line with current literature (Riches et al., 2019). As some individuals are particularly sensitive to cybersickness, it could have been useful to assess individual factors (e.g., susceptibility to motion sickness) that contribute to these symptoms. This would help determine whether the symptoms are due to the task or to participant-specific characteristics, and how they affect the overall perception of realism and SMR maturity (Mittelstaedt, 2020; Kourtesis et al., 2024; Ramaseri Chandra et al., 2022). Further, this could help determine whether a person is a good candidate for this type of assessment in the clinical context. Despite the significant association between cybersickness and task engagement, the highest-rated items on the PQ realism subscale were those assessing involvement and engagement. This suggests that, despite some cybersickness symptoms, participants remained engaged, indicating that cybersickness is unlikely to be a major concern for user engagement in the So-Moral-VR (Wang et al., 2018; Zaidi and Male, 2018; Witmer and Singer, 1998). While cybersickness may reduce the perceived realism of the task, it most likely affects specific elements such as navigation, movement and interaction, rather than overall engagement.

### Quantitative support for veridicality of the So-Moral-VR task

The positive associations between the SMR score and prosocial behavior supports the veridicality of the task in typically developing youth (i.e., the extent to which test scores correlate with other measures that are predictive of real-world task performance).

#### Association between socio-moral reasoning and behavior

The association between SMR measures in VR and prosocial behavior was statistically significant, with a small effect size, suggesting some degree of veridicality for the task. This result aligns with previous work showing similar associations. Li et al. (2025) found that moral sensitivity (the ability to recognize and interpret moral issues based on one’s experiences and understanding) measured in 567 college students positively predicted prosocial behavior. However, it should be noted that no significant associations were found with negative behavioral manifestations such as conduct or peer relationship problems. One possible explanation is that the participants had a typical course of development, therefore it was not expected that they would present clinically significant behavior problems. Associations between social cognition and behavior are less likely in typically developing populations since there is less variability and ceiling effects (Lincoln et al., 2021). Future work could seek to examine these associations in clinical populations at risk for social behavior difficulties. Alternately, such an association may be difficult to detect because of social desirability bias in questionnaire-based assessments of social function (Crowe et al., 2011; Frankel and Feinberg, 2002).

### Low susceptibility to social desirability bias in the So-Moral-VR task

Importantly, no association was found between the two So-Moral-VR scores (SMR, SMDM) and social desirability, suggesting that design choices – such as using the mobile phone interface external control of verbal interventions—were probably effective in limiting social desirability bias. However, the impact of this design change was not measured directly. Nevertheless, this innovative feature may not eliminate the social desirability effect entirely, as the user is still aware that an adult is nearby. Future research could quantitatively compare the use of a human examiner versus the cell phone method, as well as study the effect of other design choices and features on social desirability. For example, a self-administered and fully anonymous remote assessment could be considered, as well as using fully automated transcription and coding, which could encourage users to self-disclose or respond more honestly.

### Applications to the study and management of clinical and social conditions

Many neurodevelopmental conditions, acquired brain injuries, mental health problems, and social disadvantage are associated with social difficulties (Anderson et al., 2023; Beauchamp et al., 2013; Kenny and Lennings, 2007; Beauchamp et al., 2019; Fong and Iarocci, 2020) that can in part be explained by underlying deficits in social cognition. As a first step to documenting the ecological validity of the So-Moral-VR tool, only typically developing children and adolescents were recruited for the current study. Future work should determine whether the results apply to clinical populations of interest that may be at-risk for social difficulties and to evaluate other psychometric constructs, such as sensitivity, by comparing performance on the VR task between typically developing and clinical populations. It is possible that the task may require adaptations to ensure comprehension or other accessibility features in such populations.

### Strengths and limitations

Few reports that introduce novel psychological and neuropsychological assessment tools provide detailed background information on design and development decisions that lead to the final content and structure of the tasks. Further, there is a lack of formal psychometric validation and comprehensive evaluation of ecological validity within VR tasks (Neveu et al., 2025). This study aimed to address these gaps in relation to an innovation VR measure of SMR. However, the study also has some limitations that need to be considered. Questionnaires were used to measure presence, yet this approach is sometimes criticized because participants are required to recall how they were feeling, which can be prone to recall errors (Parsons et al., 2017). To limit this, the questionnaire was completed directly after the So-Moral-VR task. Future work should use objective or neurophysiological measures to track sense of presence (Parsons et al., 2017). Similarly, measures of veridicality also relied on self-report, which may not be the most accurate representation of everyday behaviors and subject to desirability bias. Observational behavioral measures could be used to provide a more accurate measure of naturalistic behavior. Nevertheless, social cognition rating scales are reported to better predict adaptative and daily functioning than some neuropsychological tasks (Silberstein and Harvey, 2019). Finally, the empirical analyses combined two samples of participants and therefore some measures were only used in a subset of the study sample.

## Conclusion

This study presents a detailed description of the *a priori* development design principles and process for a VR-based task measuring everyday SMR in children and adolescents, and provides quantitative support for its ecological validity and low susceptibility to social desirability. The So-Moral-VR is the first VR assessment tool available for measuring SMR in the pediatric population and, as such, represents a significant step in the quantitative assessment of social cognition in children and adolescents. The findings suggest that the So-Moral-VR task is a promising tool for use in clinical and research contexts.

## Data Availability

The datasets presented in this article are not readily available because authorization to re-use the data for other studies was not included in the initial consent form signed by participants. Requests to access the datasets should be directed to miriam.beauchamp@umontreal.ca.
